# Some Unusual Conditions Found in Operating for the Radical Cure of Hernia

**Published:** 1914-03

**Authors:** Charles A. Morton

**Affiliations:** Professor of Surgery in the University of Bristol; Senior Surgeon to the Bristol General Hospital and the Hospital for Sick Children and Women


### SOME UNUSUAL CONDITIONS FOUND IN OPERATING FOR THE RADICAL CURE OF HERNIA.

BY

CHARLES A. MORTON, F.R.C.S.,

*Professor of Surgery in the University of Bristol; Senior Surgeon to the Bristol General Hospital and the Hospital for Sick Children and Women.*

IN operating for hernia with retained testis, it is not uncommon to find that the vas makes a slight excursion in the wall of the sac in front of the testis. In a case on which I operated this complication occurred to so marked a degree that the vas extended for almost three inches in front of the testis. I did not recognise that I was dealing with the vas so far in advance of the testis, and divided it on the side on which I first operated, and I think the record of the case may be a valuable caution to other surgeons in operating for this combination of hernia with retention of the testis within the abdomen.

W. T., aged 18, was admitted to the General Hospital on June 4th, 1912. The scrotum was very imperfectly developed, and there was no sign of the testicle on either side. There was then no sign of a hernia on the right side, but he said that from time to time a small swelling appeared in that inguinal region. On his left side there was a small swelling between the external ring and the top of the imperfectly-developed scrotum, which disappeared when he lay down. It did not feel like even an abnormally small testis, nor did it feel quite like a hernia. He told us that this swelling had appeared at intervals, when he was about, ever since he could remember anything of the appearance of the parts.

At the operation, on dividing the external oblique on the left side, and thus laying open the inguinal canal, the sac was at once exposed and I opened it. In its floor lay a thick fold, which looked as if it might be the cord, but it terminated about the region of the external ring, gradually shelving down to the level of the sac wall. It seemed to contain some large veins, and I thought I could feel the vas in it, but it seemed difficult to understand how the vas could be running, as it apparently did, from the testicle out through the external ring. I found the testis just inside the internal ring. It was quite small and the globus major was very loosely attached to it, and the globus minor was spread out in the sac wall. I then felt whether I could detect the vas running from the testis under the parietal peritoneum, through the obturator region down to the base of the bladder; I could quite distinctly do so. I therefore concluded that the thick fold in the floor of the sac, which was almost three inches in length, could not be the cord, and I decided to cut the sac across just beyond the testis, *i.e.* through this fold, remove the distal portion (which reached as far as the top of the imperfectly-developed scrotum), and make a covering for the testis of the remainder. The testis, with this covering, was then pushed well into the peritoneal cavity, and the internal ring closed by suturing. On examining the sac after removal, I found that it undoubtedly contained the vas and some veins of the cord. The vas had been twice divided, where it ran parallel with itself, for after leaving the testis it made a long loop in the fold in the sac wall, and then returned to the region of the internal ring to descend towards the base of the bladder. Having elucidated this puzzling condition on the left side, I could now make out the relation of the parts in the right hernial sac. It also had a fold in its floor, but not such a thick one as on the left side, and the testis also lay on this side just inside the internal abdominal ring. On separating the sac from the inguinal canal and holding it against the light, I could trace the vas coming from the end of the spread-out globus minor, making a long loop in the sac wall, and returning to the internal ring, from which I could follow it with my finger, passing down to the region of the bladder under the peritoneum, as on the other side. I found it was impossible to remove more than quite the distal end of the sac without damaging the vas, and I had first to return the testis into the abdomen, and then the remainder of the sac after it, closing the internal ring by passing a purse string suture round it, partly through the peritoneum and partly through the loose subperitoneal connective tissue. It was quite impossible to dissect up the very thin peritoneum off the spread-out globus minor and elements of the cord. The peritoneum was extremely thin, and these structures were incorporated with it. In operations for hernia in children the peritoneum of the sac is often extremely thin and difficult to separate from the elements of the cord: this can generally be done sufficiently to get a sac neck to tie, but in this case it would have been quite impossible.

It is a much-debated question whether such imperfectly-developed testes as this patient had, lying within the peritoneal cavity, are capable of secreting spermatozoa, and therefore it is quite possible he is not any the worse for the removal of the left vas, but as it seemed just possible they might form spermatozoa, I did not like to remove it on the other side as well, though if I had removed the sac on this side also it would have made it a more thorough operation for the cure of the hernia. On both sides I dealt with the inguinal canal by the ordinary Bassini's method, and I was of course able to completely close each external ring.

I am not quite certain what it was I felt before operation on the left side. It could not have been the testis, for it could not come near the external ring. I am inclined to think it was the sac, with the very thick fold already referred to, in it.

The only reference I have been able to find to anything like the condition I found in this case is in Mr. McAdam Eccles's book on *The Imperfectly Descended Testis*. He says (page 81): "When the testis has remained behind in the abdomen, and a peritoneal pouch has descended into the canal, it occasionally happens that the vas deferens, or even the epididymis have also passed into the passage, and may be felt as cords lying posterior to the hernial sac."

This is what happened in my case, but what seems to me the point of special interest in my own case was the great length to which the loop of cord extended in the sac wall in front of the testis. A very misleading feature of the condition is the way in which the vas can be traced running from the region of the retained testis down under the parietal peritoneum towards the base of the bladder, so that one is apt to think a fold in the floor of the sac a considerable distance in front of the testis cannot contain the vas, whereas the vas is making a long loop in the fold and returning to the region of the testis, and then descending in the usual position under the parietal peritoneum to the base of the bladder. It seems to me that a description of the details of my own case may be a help to any surgeon who comes across a similar condition.

In operating for radical cure of hernia, it is not uncommon to find masses of fat in the inguinal canal, associated with the presence of a hernial sac. They are protrusions of the sub-peritoneal fat, but may only be connected with it by a narrow pedicle. But in two cases in which I recently operated and found such masses of fat, there was no true sac. In one case such a mass of fat protruded from the external ring, and in the other through the crural canal.

There is a paper in the *Annals of Surgery* for February of last year (1913) by Dr. Louis Freedman, of New York, on "Hernia Adiposa," as he calls this condition, with a record of one case. In this case the fat hernia was not reducible, and simulated an irreducible omental hernia. In one of my own cases it was reducible, but the piece of fat of course reduced only into the inguinal canal, yet it seemed to reduce as completely as any small ordinary hernia may. It is quite likely that in cases like my own, in which there is no true sac, in the course of time a process of peritoneum may be drawn out by the descending lump of fat, and then the more common form of combined sac and fatty mass found. The growth of these fatty masses must in all cases open up the inguinal canal ready for the descent of a hernia, even if they do not actually drag down a peritoneal sac. In performing a radical cure of hernia, in which masses of fat are present in addition to a true sac, it is very important to remove them from the inguinal canal. If in operating for cure of hernia such a fatty mass as I found in these two cases appeared the only abnormal structure present, it would be necessary to examine with great care that no hernial sac, however small, was buried in it, and also that it did not contain a very small portion of the bladder.

Small protrusions of subperitoneal fat, without a definite peritoneal sac, not rarely occur through holes in the aponeurosis at the linea alba, but a fat hernia is not common in the inguinal or femoral regions, and though they were fully described by Mr. J. Hutchinson as long ago as 1886 in the *Transactions of the Pathological Society* (Vol. 37), there is very little information about them in some books on hernia, or in the ordinary text books of surgery. Mr. Hutchinson says it is almost impossible to distinguish them from omental hernia, and in several other cases besides mine operation has been performed under the impression that the swelling was an ordinary hernia. No doubt operation is good treatment, for as Mr. Hutchinson points out, the descent of a lump of fat is likely to drag down a true hernial sac in time, and the patient could not tell when that had occurred. Mr. Hutchinson very well describes the usual appearance of these fatty masses. He says, "The capsule in some cases very closely resembles peritoneum, the chief difference is, that it usually gives off many septa which lie between the contained lobules of fat."

A man, S. B., aged 50, was sent to me at the General Hospital in February, 1913, with a good-sized hydrocele which had been present for six years, and reached up to the external ring. When it was tapped a soft protrusion the size of a walnut could be made out at the external ring on coughing, and I diagnosed a hernia which had before been kept up by the hydrocele acting as a truss. On February 28th I operated on the hydrocele, and then laid open the inguinal canal for the radical cure of hernia. There was no true hernial sac, but a loose elongated mass of fat occupied the canal, and lay by the side of the cord. It formed a soft lipoma, and evidently grew from the subperitoneal fat at the region of the internal ring. The external ring was enlarged. I removed this fatty mass, and then found a similar one protruding from the subperitoneal fat in the iliac fossa, under the cord. It was divided into two lobes by the passage of the deep epigastric artery over it. I tucked this mass more deeply down into the iliac fossa, and then united the internal oblique and transversalis to Poupart's ligament as in Bassini's operation, and in uniting the external oblique narrowed the external ring.

I have operated on another case for radical cure of inguinal hernia, a man 48 years of age, in which I found a very small sac combined with several well-marked fatty masses. So small was the sac, that it seemed to me it could never have protruded through the external ring, but that the fatty masses did so, and had dragged down the small sac in their descent.

A woman, G. V., aged 27, was sent to me at the General Hospital in September last for radical cure of double inguinal hernia. I thought there was also a very small femoral hernia on the right side. I operated on each inguinal hernia, and on passing my finger down below Poupart's ligament on the right side into the region of the saphenous opening, I found a femoral hernia the size of a small marble. There was no true sac, and it was only a protrusion of subperitoneal fat. I exposed its neck from the deep aspect, through the incision just above Poupart's ligament made for the cure of the inguinal hernia, and then drew the protrusion of fat back through the crural canal, and carefully searched for a small sac, but found none. But I closed the deep aspect of the crural ring in the usual manner when operating for radical cure of femoral hernia by the inguinal method.[1]

The third unusual condition I have met with when operating for the cure of hernia, which I will describe in this paper, is the formation of a cyst on the round ligament, at the external ring, associated with a very narrow track of open peritoneum extending along the round ligament. The case was a very puzzling one before operation. In a woman, A. J., aged 30, who came under my care at the General Hospital in March, 1913, there was a small fluctuating swelling just over the external ring. It did not in the least feel like a hernia, but the patient stated that it had been decidedly larger than it was at the time of examination, so that the suspicion was raised as to whether it might not be associated with the descent of a hernia at times. It was found at the operation to be a cyst in the end of the round ligament, and there was a very narrow open process of peritoneum extending down the inguinal canal to it, but the lumen of the process was too small for the descent of any

[1] Since this paper was written I have operated on a case of inguinal hernia in a woman, and found a lump of subperitoneal fat incorporated with the distal end of the sac. The sac wall was so completely merged into the lump of fat that it is not likely the sac in its descent had pushed it before it, but rather that the rupture had started as a fat rupture, and then dragged down a true peritoneal sac.

bowel, or more than the very finest tag of omentum. Still such an open tube of peritoneum did constitute a form of hernial sac. It terminated at the cyst, and it is quite possible that the cyst may have originated in a portion of the tube, which became shut off. The cyst contained clear fluid. Such cysts are sometimes called hydroceles of the canal of Nuck.

